# Patient‐ and Clinician‐Reported Outcomes and Outcome Measures Evaluating Timing of Implant Loading in the Edentulous Maxilla: A Systematic Review of Prospective Studies

**DOI:** 10.1111/clr.14451

**Published:** 2026-02-24

**Authors:** Claudio Mendes Pannuti, Mohamed A. Hassan, Isabella Neme Ribeiro Reis, Cristina Cunha Villar, Helena Francisco, Giuseppe A. Romito

**Affiliations:** ^1^ Division of Periodontology, Department of Stomatology, School of Dentistry University of São Paulo São Paulo Brazil; ^2^ Department of Periodontology and Oral Implantology, Dental Research Division Guarulhos University – UNG Guarulhos Brazil; ^3^ Department of Oral Surgery and Implant Dentistry, Faculty of Dental Medicine Lisbon University Lisbon Portugal; ^4^ Implantology Institute Lisbon Portugal

**Keywords:** dental implants, edentulous jaw, immediate dental implant loading, patient‐reported outcome measures

## Abstract

**Aim:**

To identify patient‐reported outcomes (PROs) and clinician‐reported outcomes (ClinROs) and their outcome measures (PROMs and CROMs) used in prospective studies comparing immediate, early, and delayed implant loading protocols in edentulous maxilla patients receiving implant‐supported prostheses.

**Materials and Methods:**

Protocol‐driven electronic searches were conducted across MEDLINE, Embase, Scopus, Web of Science, Cochrane databases—January 2014 to May 2024—to identify prospective interventional and observational studies comparing immediate, early, delayed implant loading in maxillary edentulous patients. Zygomatic implants were excluded. Risk of bias was performed using Cochrane RoB 2 and ROBINS‐I, and results were reported descriptively.

**Results:**

Five studies were included, of which four compared immediate versus delayed loading and one study compared immediate versus early loading. PROMs like visual analog scale (VAS) tool (reported in 80%) to evaluate 7 PROs: pain, overall satisfaction, comfort, speech, masticatory function, esthetics, and self‐esteem. Three studies reported Oral Health Impact Profile (OHIP‐19, OHIP‐20), McGill Pain Questionnaire, and condition‐specific instruments assessing two outcomes: chewing ability and muscular activity during oral functions. ClinROs varied considerably between studies, demonstrating significant methodological diversity. ClinROs included implant stability, accuracy, occlusal parameters, periodontal health metrics, surgical outcomes, and esthetic evaluation via VAS.

**Conclusion:**

PROs were predominantly assessed using VAS to evaluate patient satisfaction, pain, function, and esthetics, followed by the OHIP Questionnaire for quality‐of‐life assessment in immediate versus early/delayed loading protocols. ClinROs showed no standardized approach, creating significant heterogeneity in the reported outcomes. Standardization of assessment methods and reporting of PROMs/CROMs is needed to optimize analyzing patient‐centered outcomes.

## 1 Introduction

The rehabilitation of edentulous maxillary arch with removable and fixed implant‐supported prostheses has become an increasingly common treatment modality in contemporary dental practice (Capelli et al. 2007; Kern et al. 2016). One of the critical considerations in implant therapy is the timing of loading, which refers to the point at which the prosthetic restoration is connected to the implants following their surgical placement (Esposito et al. 2007). The traditional approach involved a delayed loading protocol (DL), wherein the implants were allowed to heal for an extended period, typically more than 2 months, before prosthesis connection and functional loading (Brånemark et al. 1977; Esposito et al. 2007). However, more recent strategies, such as immediate and early loading protocols, have gained popularity due to their potential benefits, including reduced treatment duration, improved patient acceptance, and earlier restoration of function and esthetics (Kim et al. 2018; Nicolau et al. 2019).

Immediate loading (IL) involves connecting the prosthesis to the implants within 1 week of implant placement, while early loading (EL) is defined as loading between 1 week and 2 months after implant insertion (Schimmel et al. 2014). These accelerated protocols have been facilitated by advancements in implant surface technologies, surgical techniques, and prosthetic designs, which aim to enhance implant stability and osseointegration (Nicolau et al. 2019; Smeets et al. 2016). Nonetheless, the optimal timing of loading remains a subject of ongoing debate, with considerations regarding implant survival, marginal bone loss, and patient‐reported outcome measures (PROMs) (Abdunabi et al. 2019).

Patient‐reported outcomes (PROs) provide valuable insights into patients' perspectives on various aspects of their treatment, including satisfaction, quality of life, pain, and functional outcomes (Duong et al. 2022; Feine et al. 2018). PROMs are the structured tools, like questionnaires or scales, that capture these PROs directly from patients, without any alterations or interpretations by clinicians or others, ensuring a standardized assessment of the patient's perspective (Weldring and Smith 2013). Conversely, clinician‐reported outcomes (ClinROs) offer assessments made by healthcare professionals based on clinical observations and objective tests, requiring specialized training to accurately interpret signs, behaviors, or other manifestations related to a condition (FDA Glossary 2018). ClinRO includes subjective and objective outcomes such as clinical outcomes, treatment feasibility, esthetic outcomes, and overall satisfaction with the procedure (Cosyn et al. 2017; Powers et al. 2017). Clinician‐reported outcome measures (CROMs) include reports of specific clinical findings or events and/or the use of rating scales (FDA Glossary 2018).

PROMs and CROMs are increasingly recognized as essential components in evaluating the PROs and ClinROs in dental implant treatments (McGrath et al. 2012). The Implant Dentistry Core Outcome Set and Measurement (ID‐COSM) has emphasized that patient satisfaction and comfort are one of the mandatory outcome domains to assess in all implant trials, while quality of life assessment becomes mandatory in specific conditions/populations being evaluated (Tonetti et al. 2023). While ID‐COSM provides valuable foundational guidance on essential outcome domains in implant clinical trials, significant gaps remain in its practical application. Specifically, there is a need to examine how these outcomes are measured and reported in studies investigating different loading protocols for edentulous maxilla, an aspect that falls outside the primary scope of ID‐COSM's framework and existing evidence syntheses (Tonetti et al. 2023).

While several systematic reviews have examined the clinical and radiographic outcomes of different implant loading protocols (Gallucci et al. 2009; Papaspyridakos et al. 2014), a comprehensive evaluation of the PROs and ClinROs utilized in studies investigating the timing of loading for edentulous maxillary patients is lacking (Abdunabi et al. 2019). Such an assessment is crucial to inform evidence‐based decision‐making, facilitate patient‐centered care, and identify areas for further research and outcome measure development.

Therefore, the primary objective of this systematic review was to identify the PROs and ClinROs that have been employed in studies evaluating the timing of loading in implant treatments in the upper jawbone. Additionally, this review aimed to examine the measures of assessment (PROMs and CROMs) used to report these outcomes, providing a comprehensive overview of the current evidence and highlighting potential gaps or areas for improvement in outcome reporting.

## 2 Materials and Methods

### 2.1 Protocol and Registration

This systematic review was reported in accordance with the guidelines of the Preferred Reporting Items for Systematic Review and Meta‐Analyses (PRISMA 2020) (Page et al. 2021) (checklist available on Table S1). This systematic review was conducted following a preregistered protocol approved by the organizing committee of the Global Consensus for Clinical Guidelines (GCCG). We focused on prospective studies to ensure standardized reporting of outcome measures and minimize potential recall bias in PROs and ClinROs assessment. The protocol was prospectively registered at the PROSPERO database (CRD42024519360).

### 2.2 Focused Questions and PICOS Outline

#### 2.2.1 Question 1

(1) In patients with an edentulous maxilla undergoing dental implant treatment with varying timings of loading, what PROs and ClinROs have been utilized?

#### 2.2.2 Question 2

(2) In patients with an edentulous maxilla undergoing dental implant treatment with varying timings of loading, what measures of assessment (PROMs and CROMs) have been employed to report PROs and ClinROs?

Although the present review addresses two focused questions, it explores complementary aspects of the same population, intervention, and outcomes: Question 1 focuses on the identification of reported PROs and ClinROs, while Question 2 addresses the measures used to measure these outcomes (PROMs and CROMs).

#### 2.2.3 PICOS Outline

- Population (P): Patients with an edentulous maxilla and needing a fixed or removable implant‐supported prosthesis
- Intervention (I): Implant therapy with one of the following loading protocols:
  ○ Immediate loading: prosthesis connection within 1 week post‐implant placement.
  ○ Early loading: prosthesis connection between 1 week and 2 months post‐implant placement.
  ○ Delayed loading: prosthesis connection after 2 months post‐implant placement.
- Comparison (C): The following comparisons were considered:
  ○ Immediate versus delayed loading.
  ○ Immediate versus early loading.
  ○ Early versus delayed loading
- Outcomes (O): Primary outcomes: PROs/PROMs; Secondary outcomes: ClinROs/CROMs.
- Study (S): Prospective interventional and observational studies.

### 2.3 Eligibility Criteria

#### 2.3.1 Inclusion Criteria

- Prospective comparative studies directly comparing outcomes between at least two different implant loading protocols (immediate vs. delayed, immediate vs. early, or early vs. delayed loading) with clearly reported timing of loading after implant placement, in accordance with a preregistered protocol approved by the consensus committee.
- Prospective interventional and observational studies (randomized controlled trials, non‐randomized clinical trials, cohort studies, case series with at least 10 cases).
- Patients with a completely edentulous maxilla or those scheduled for complete maxillary tooth extraction, who would receive implants and full‐arch fixed or removable implant‐supported prostheses.
- Studies evaluating both PROs/PROMs and/or ClinROs/CROMs.
- Studies published within the past 10 years (from 2014 to present) focus on recent practices.
- Studies in English language promote consistency in data extraction and analysis, as all authors contributing to the special volume are fluent in English, which is also the predominant language in this field.

#### 2.3.2 Exclusion Criteria

- Retrospective studies, cross‐sectional studies, case reports, in vitro, and preclinical studies.
- Studies involving implants that had already been placed prior to study initiation.
- Studies involving implants placed in the pterygoid or zygomatic bones.
- Studies comparing other interventions at a single time point rather than loading protocols, single‐arm studies where all participants receive early, immediate, delayed implant prosthetic loading, in alignment with the guidance provided by the consensus committee that commissioned this review.

### 2.4 Search Methods

An electronic literature search was conducted up to May 2024, across multiple databases, including MEDLINE (PubMed), Embase, Scopus, Web of Science, and Cochrane Central Register of Controlled Trials. The search strategies for each database were developed using key terms related to the focused question and PICOS criteria. Search strategies are reported in Table S2.

### 2.5 Study Selection and Data Collection

Two independent reviewers (M.A.H. and I.N.R.R.) screened the titles and abstracts of the studies identified through the search strategy based on the predefined eligibility criteria using the Rayyan platform (Ouzzani et al. 2016). Any disagreements were resolved through discussion or by consulting a third reviewer (C.M.P.). The inter‐rater reliability test (Cohen's Kappa) was calculated to measure the agreement between the two reviewers by applying a pilot test for 10% of the retrieved studies. The full texts of potentially eligible studies were then evaluated by the same reviewers, and detailed records were maintained documenting the reasons for excluding studies. The included studies underwent data extraction by the same authors. Data were extracted based on the following characteristics:

1. Study characteristics: country, study setting, study design, number of arms, funding, primary outcome.
2. Participants: number of patients (included/baseline/last follow‐up), sex (male/female), mean age (years), maximum follow‐up (years).
3. Characteristics of implant and prosthesis: timing of loading, implant's location, number of implants, number of implants per patient, implant type, type of prosthesis (fixed or removable), prosthesis details (provisional/definitive prothesis), mandibular condition.
4. Outcomes: PROs, ClinROs, PROMs, and CROMs tools, questionnaire, questionnaire reference, analysis metrics, time points, clinician training/calibration.

### 2.6 Risk of bias

The risk of bias assessment utilized different tools based on the study design. For randomized clinical trials, we used the Cochrane Collaboration's Tool for Assessing Risk of Bias (RoB 2) (Sterne et al. 2019), assigning judgments of “Low risk of bias,” “Some concerns,” or “High risk of bias” to each of the five domains: bias arising from the randomization process, bias due to deviations from intended interventions, bias due to missing outcome data, bias in measuring the outcome, and bias in the selection of the reported result.

Non‐randomized clinical trials was evaluated using the Risk of Bias in Non‐randomized Studies of Interventions (ROBINS‐I) tool (Sterne et al. 2016), endorsed by the Cochrane Collaboration. This tool assesses bias related to confounding, selection, exposure classification, deviations from intended interventions, missing data, outcome measurements, and selection of reported results, with categories including “Low risk of bias,” “Moderate risk of bias,” “Serious risk of bias,” “Critical risk of bias,” and “No information.”

Two reviewers (M.A.H. and I.N.R.R.) independently appraised the methodological quality and risk of bias of the included studies. The *Robvis* tool was used to create risk‐of‐bias plots (McGuinness and Higgins 2021).

### 2.7 Data Synthesis

PROs and ClinROs and their assessment measures (PROMs and CROMs) were identified and categorized while qualitative data synthesis was not feasible. Data was analyzed using STATA version 16.1 (StataCorp, College Station, TX, USA) to calculate frequencies and percentages of different outcome measures.

## 3 Results

### 3.1 Search and Screening

**FIGURE 1:**
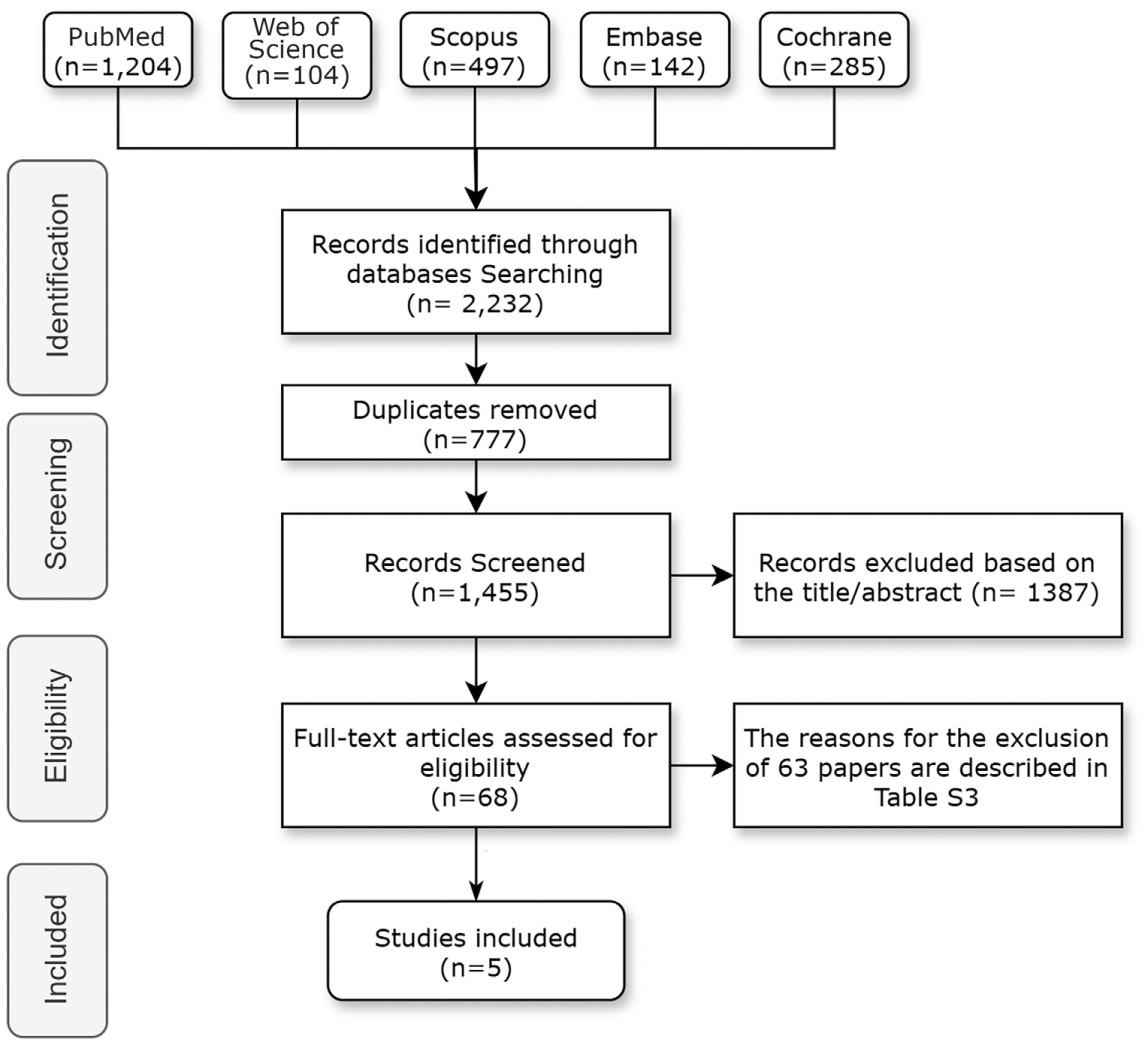
Flowchart.

The literature search across multiple databases initially identified 2232 records. After removing 777 duplicates, 1455 records were screened based on titles and abstracts, leading to the exclusion of 1387 records. The full texts of the remaining 68 potentially eligible studies were assessed, resulting in the final inclusion of four randomized controlled trials (RCTs) and one non‐randomized controlled trial (n‐RCT) that met the eligibility criteria (Figure 1). The study demonstrated a high level of inter‐examiner agreement, with a kappa (*κ*) value of 0.81%. Excluded articles with reasons are summarized in Table S3.

### 3.2 General Characteristics of the Included Studies

The characteristics of the included studies are presented in Table 1. All trials were based at university settings and employed a two‐arm, parallel‐group randomized controlled design for four studies and one study with a non‐randomized controlled study design. The studies included a total of 121 patients and analyzed 724 implants with different loading protocols. Sample sizes ranged from 15 patients (Bernard et al. 2019; Vercruyssen et al. 2016) to 34 patients at baseline (Montero et al. 2021). Eighty percent of studies received external funding support (Bernard et al. 2019; Marković et al. 2022; Montero et al. 2021; Vercruyssen et al. 2016) except for Peñarrocha‐Oltra et al. (2014). Participants' mean age spanned from 45 to 71 years in the immediate loading groups and 49–67 years in the delayed loading arms. Follow‐up duration was variable, with a minimum of 1 week in Vercruyssen et al. (2016) and a maximum of 2 years in Bernard et al. (2019). The primary outcomes investigated included bone loss (Bernard et al. 2019), implant stability (Marković et al. 2022), and PROMs were reported as secondary outcomes. While three studies (60%) reported PROs as a primary outcomes as follows: patient satisfaction and postoperative morbidity (Peñarrocha‐Oltra et al. 2014), patient‐reported outcomes (Vercruyssen et al. 2016), and functional and subjective outcomes of implant‐supported rehabilitations (Montero et al. 2021).

**TABLE 1:** Characteristics of the included studies.

Study	Marković et al. (2022)	Montero et al. (2021)	Bernard et al. (2019)	Vercruyssen et al. (2016)	Peñarrocha‐Oltra et al. (2014)
Country	Serbia	Spain	Belgium	Belgium	Spain
Study setting	University	University	University	University	University
Study design	RCT	RCT	RCT	RCT	n‐RCT
Number of arms	Two	Two	Two	Two	Two
Funding	Institute Straumann AG	Cátedra Extraordinaria: Implantoprótesis y Oclusión” of the University of Salamanca	Dentsply Sirona provided oral implants, prosthetic materials, and stereolithographic guides	Oral implants by DENTSPLY Implants. Stereolithographic guides by the Materialise Dental Company	No
Number of patients (included/baseline/last follow‐up)	24/24/24 IL (12/12/12) EL (12/12/12)	34/34/27 IL (16/16/11) DL (18/18/16)	15/15/15 IL (7/7/7) DL (8/8/8)	15/15/15 IL (7/7/7) DL (8/8/8)	30/30/29 IL (15/15/14) DL (15/15/15)
Sex (male/female)	IL (4/8) EL (1/11)	IL (9/7) DL (10/8)	IL (5/2) DL (7/1)	IL (5/2) DL (7/1)	IL (7/7) DL (6/9)
Mean age (years)	IL (61.25 ± 7.47) EL (60.0 ± 5.41)	IL (62.8 ± 11.9) DL (67.0 ± 7.8)	IL (45–71) DL (49–70)	IL (45–71) DL (49–70)	IL (53.1 ± 10.6) DL (57.6 ± 8.8)
Maximum follow‐up	6 weeks	1 year	2 years	7 days	12 months
Primary outcome	Primary and secondary implant stability	Functional and subjective outcomes of implant‐supported full‐arch hybrid rehabilitations made of PEEK‐NFC	Bone loss	Accuracy (deviation between planned and placed implant position) and patient‐centered outcome measures	Patient satisfaction and postoperative pain and swelling

Abbreviations: DL, delayed loading; EL, early loading; IL, immediate loading.

All studies involved implant placement in the maxilla, with the number of implants ranging from 6 to 8 per patient. Various implant systems were used, including Ankylos (Dentsply Sirona), Bone Level Tapered SLActive (Straumann), Kohno SP (Sweden & Martina), and others. All studies included patients with sufficient bone volume and excluded cases requiring major bone augmentation procedures prior to implant placement. While two studies reported performing minor augmentation procedures when necessary to address peri‐implant defects or gaps at the time of implant placement (Marković et al. 2022; Peñarrocha‐Oltra et al. 2014). Both fixed and removable implant‐supported prostheses were evaluated, with some studies using provisional prostheses initially and definitive prostheses at later follow‐up timepoints. Implants and prostheses characteristics used in the included studies are presented in Table 2.

**TABLE 2:** Characteristics of the implants and prosthesis.

Study	Marković et al. (2022)	Montero et al. (2021)	Bernard et al. (2019)	Vercruyssen et al. (2016)	Peñarrocha‐Oltra et al. (2014)
Timing of loading	IL/EL	IL/DL	IL/DL	IL/DL	IL/DL
Implant's location	Maxilla	Maxilla and mandible	Maxilla	Maxilla	Maxilla
Number of implants (IL/DL)	Baseline (72/72)	Baseline (total = 210)	Baseline (42/48) Analyzed (42/48) Lost (0/1)	Baseline (42/48) Last follow‐up (42/47) Lost (0/1)	Baseline (94/99)
Number of implants per patient	6	6.3 ± 2.3	6	6	6‐8
Implant type	Bone Level Tapered SLActive (Straumann)	NR	Ankylos implants (Dentsply Sirona)	Ankylos implants (DENTSPLY Implants)	Kohno SP implants (Sweden and Martina)
Type of prosthesis (fixed or removable)	Fixed full‐arch dentures	Fixed hybrid screw retained	Fixed (Screw‐retained)	Hybrid detachable prosthesis‐ screw retained	Fixed full arch
Prosthesis details (provisional/definitive prothesis)	Provisional for immediate loading group and definitive after 6 weeks for both groups	Provisional fixed rehabilitation made of PMMA for IL group, definitive PEEK‐NFC hybrid rehabilitation for both groups	Definitive prosthesis	Definitive prosthesis	Provisional immediately loaded fixed prosthesis vs. provisional removable denture (control group)
Mandibular condition (IL/DL)	NR	fixed implant supported prosthesis (4/6) Fixed implant supported prosthesis, fixed tooth‐supported dentures/Natural teeth (12/9) Partial denture (0/3)	The mandible can have any kind of dentition if a well‐distributed contact relationship with the new prosthesis in the maxilla can be established	Any kind of dentition in mandible if well‐distributed occlusal contact relationship could be established	Natural or fixed tooth‐supported (6/7) Fixed metal‐acrylic resin implant‐supported (4/3) Fixed metal‐ceramic implant‐supported (2/4) Removable implant‐supported (2/1)

Abbreviations: DL, delayed loading; EL, early loading; IL, immediate loading; NR, not reported.

### 3.3 Risk of Bias

**FIGURE 2:**
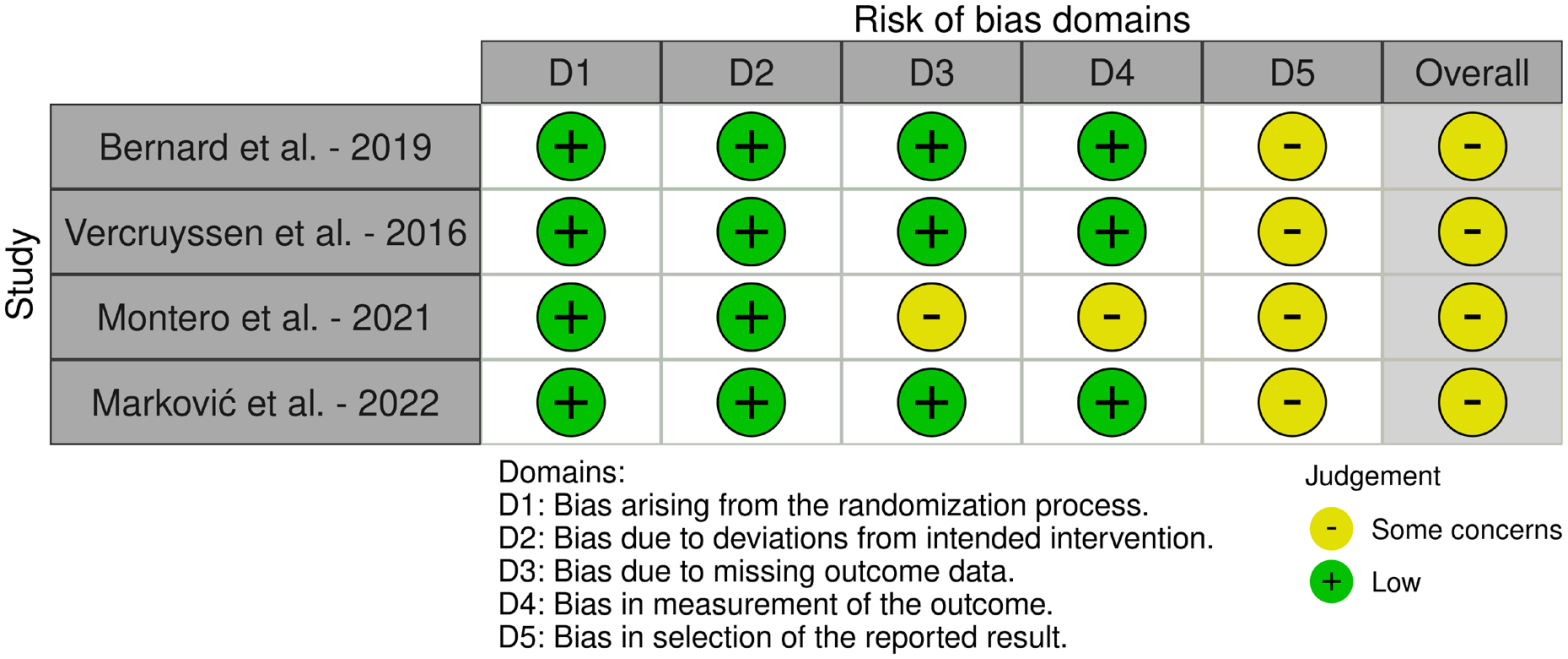
Risk of bias assessment for RCTs.

**FIGURE 3:**
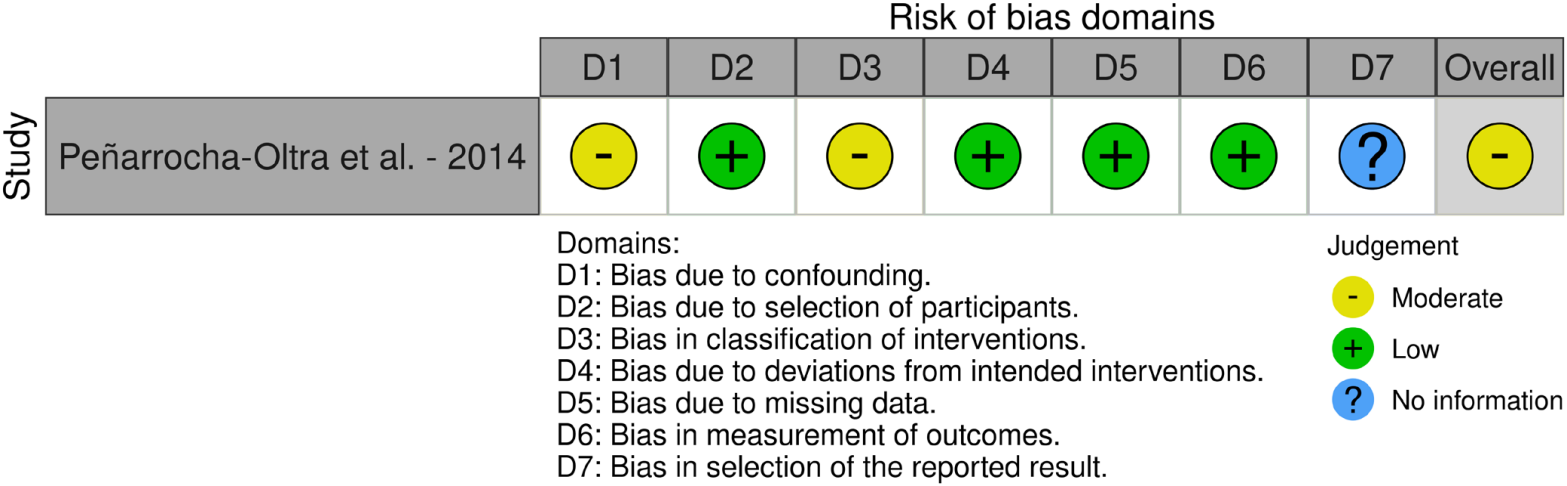
Risk of bias assessment for n‐RCT.

The risk of bias assessment for the included RCTs is summarized in Figure 2. Four studies were assessed as having a low risk of bias across most domains and overall some concerns (Bernard et al. 2019; Marković et al. 2022; Montero et al. 2021; Vercruyssen et al. 2016). While one n‐RCT reported a moderate risk of bias (Peñarrocha‐Oltra et al. 2014) (Figure 3).

### 3.4 PROs and PROMs Assessment and Implementation

**FIGURE 4:**
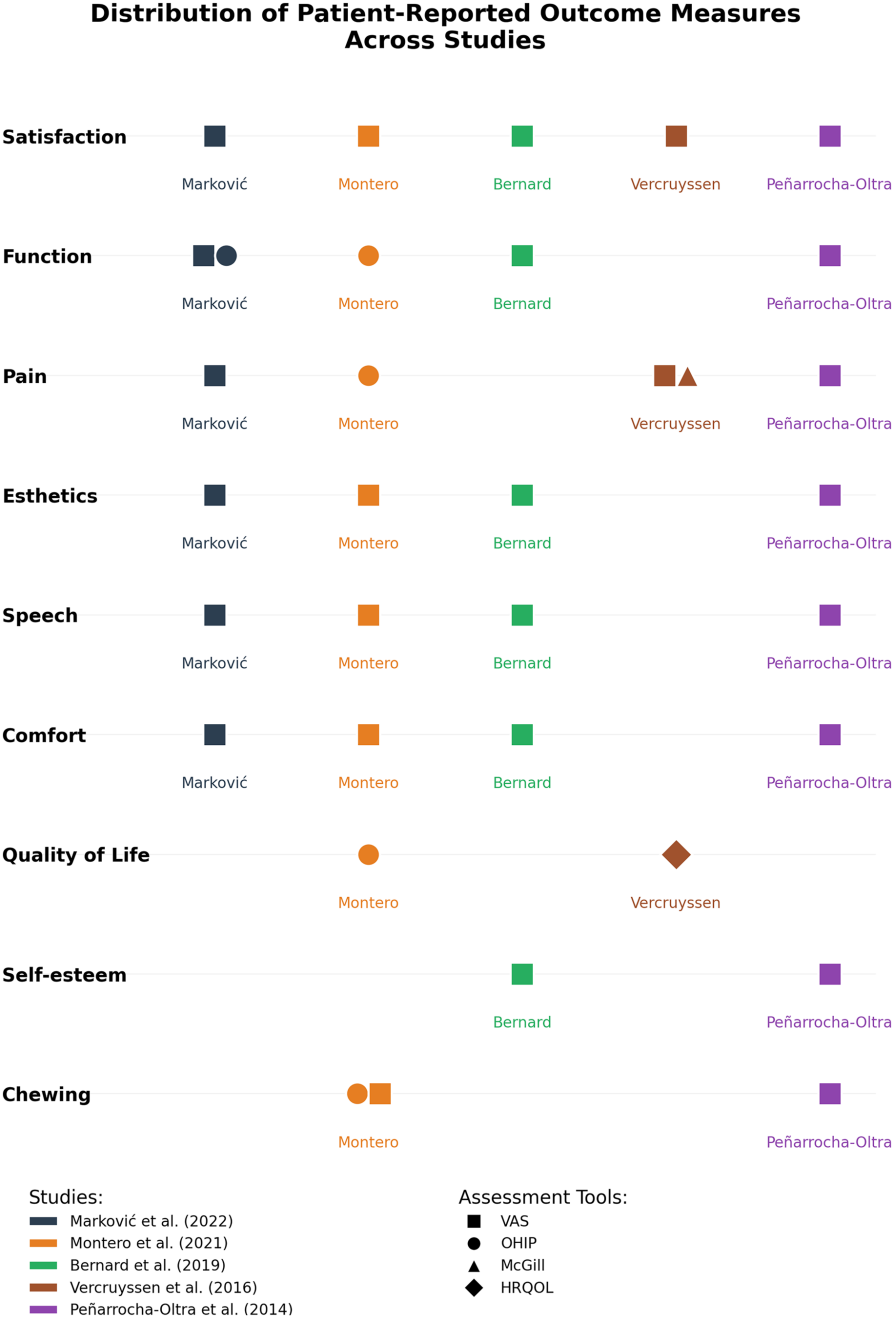
Distribution of patient‐reported outcome measures and assessment methods across studies.

The analysis of PROs utilized across studies revealed a variety of measurement instruments, as shown in Table 3 and Figure 4. The visual analog scale (VAS) was the most frequently used instrument, employed in 80% of the studies (Bernard et al. 2019; Marković et al. 2022; Peñarrocha‐Oltra et al. 2014; Vercruyssen et al. 2016). More comprehensive, validated tools included the OHIP questionnaires, with studies using either OHIP‐19 (Marković et al. 2022) or OHIP‐20 (Montero et al. 2021) to assess oral health‐related quality of life. Specialized measures were also identified, such as the McGill Pain Questionnaire (MPQ‐DLV) and HRQOL‐15, implemented by Vercruyssen et al. (2016) for detailed pain and quality of life evaluations.

**TABLE 3:** PROs and PROMs assessment.

Study	Marković et al. (2022)	Montero et al. (2021)	Bernard et al. (2019)	Vercruyssen et al. (2016)	Peñarrocha‐Oltra et al. (2014)
PROMs (questionnaires/tools)	OHIP‐19 VAS	OHIP‐20 Self‐reported chewing ability Retrospective self‐assessment of the wellbeing change by global transitional items Masticatory performance measured by chewingApp	VAS	MPQ‐DLV HRQOL‐15 VAS	VAS
Detailed PROs	OHIP‐19 (functional limitation, physical pain, psychological discomfort, physical disability, psychological disability, social disability, and handicap) Patient satisfaction using VAS (function, esthetics, discomfort/pain)	OHIP‐20 (functional limitation, pain, psychological discomfort, physical disability, psychological disability, social disability, and handicap) Chewing ability Self‐rated satisfaction (global, esthetic, chewing). Oral functions Masticatory performance	Using VAS, participants scored prosthesis retention, comfort, speech, function, esthetics, self‐esteem, and the feeling of “being my own teeth.” The VAS consisted of 20 questions Examples were “Can you eat well with your current prosthesis?”, “Can you talk well?”, “Are you content with the current situation?”	Using McGill Pain Questionnaire (MPQ‐DLV), Health‐Related Quality of Life (HRQOL), and VAS to report Pain intensity, pain quality, quality of life, pain, swelling, tolerability of procedure	VAS to report overall satisfaction, esthetics, chewing function, speech function, comfort, self‐esteem, ease of cleaning, treatment duration
Analysis metrics	Change from baseline, mean ranks	OHIP‐20 summary score, masticatory performance mean change from baseline	Mean VAS scores	Number of words chosen (NWC‐T), pain rating index (PRI‐T), HRQOL index, VAS scores	Mean, median, standard deviation, range values for VAS scores
Time points	Prior to implant placement, 1 month after definitive prosthetic restoration	Pretreatment, posttreatment (1 year)	1 year, 2 years after prosthesis placement	Daily for 7 days	Baseline, 3 months, 12 months after implant placement
Examiner training/calibration	NR	NR	NR	NR	Single trained clinician

Abbreviation: NR, not reported.

Regarding assessment measures and implementation strategies, studies employed diverse approaches in both timing and domain coverage. VAS was implemented to assess multiple domains including satisfaction, pain, function, and esthetics, while OHIP questionnaires provided structured assessments across domains such as functional limitations, physical pain, psychological discomfort, and social disability. Assessment timing varied considerably, from daily evaluations in the immediate post‐procedure period (Vercruyssen et al. 2016) to long‐term follow‐ups extending up to 2 years (Bernard et al. 2019). Different analysis metrics were used: Bernard et al. (2019) used mean VAS scores, Montero et al. (2021) employed OHIP‐20 summary scores, while Vercruyssen et al. (2016) utilized specific metrics such as number of words chosen (NWC‐T) and pain rating index (PRI‐T). This variation in assessment measures and timing reflects the lack of standardization in PROMs implementation across implant loading studies. References for PROMs reported on each study and validation information are summarized in Table S4.

### 3.5 ClinROs and CROMs Assessment and Implementation

Four of the five included studies reported 12 ClinROs and their assessment measures (CROMs) (Marković et al. 2022; Montero et al. 2021; Bernard et al. 2019; Vercruyssen et al. 2016). Each study evaluated distinctly different outcomes without any consensus on specific ClinROs or standardized assessment methods between the studies Table 4.

**TABLE 4:** ClinROs and CROMs assessment.

	Marković et al. (2022)	Montero et al. (2021)	Bernard et al. (2019)	Vercruyssen et al. (2016)
ClinROs	Implant stability Insertion torque Clinician esthetic satisfaction Surgical and prosthetic complications	Occlusal force (contact area, average pressure, maximal pressure, and occlusal load) Electromyography (EMG) activity	Bone loss Probing depths BoP Plaque index	Accuracy of implant placement Surgical time
CROMs used	Implant stability measured by Resonance frequency analysis (Osstell & Penguin) Insertion torque by torque wrench Clinical assessment of complications Clinician esthetic satisfaction by VAS for Abutment visibility, papilla appearance and presence, esthetic characteristics of restorations, presence of pink ceramics, external patient appearance, speech	Occlusal force/area and the maximal voluntary occlusal force measured by PRESCALE EMG activity on both masseters and temporalis muscles measured by MYOMED_932TM device	Radiographic measurements by ImageJ software Periodontal parameters Clinical examination	CT matching by Mimics software CBCT assessment
Analysis metrics	Median of point estimates at each time point	Mean, standard deviation of point estimates at each time point	Point estimates at each time point	Point estimates of deviation
Time points	Implant stability (baseline; Week 6) Clinician esthetic satisfaction (at implant placement; 1 month after definitive prosthetic restoration)	Baseline 12 months	Baseline Loading 1 year 2 years	Baseline (planning) Immediately after implant placement
Clinician training/calibration	Implant stability: intrarater or interrater reliability of two assessors was tested in a pilot study with 24 implants	NR	NR	Accuracy analysis done by different investigator Intra‐/inter‐examiner variability tested

Abbreviation: NR, not reported.

Marković et al. (2022) primarily assessed implant stability using two different devices: Resonance Frequency Analysis with Osstell and Penguin RFA instruments, producing implant stability quotient (ISQ) values on a scale of 1–100. They also measured insertion torque (IT) in Ncm and clinician esthetic satisfaction using VAS. The authors performed interrater and intrarater reliability testing for implant stability measurements, with no information regarding calibration. Marković et al. (2022) incorporated clinician evaluations of esthetic outcomes through a VAS rating. Clinicians provided VAS ratings for their satisfaction with aspects such as abutment visibility, papilla appearance and presence, esthetic characteristics of the restorations, the use of pink ceramics, the external appearance of the patient, and speech. Clinician VAS ratings for esthetic satisfaction were analyzed through the median change and 95% CI.

Montero et al. (2021) focused on functional parameters using pressure‐sensitive colorimetric sheets (Dental PRESCALE) to record occlusal contact area (mm^2^), average pressure (MPa), maximal pressure (MPa), and occlusal load (Nw). They also measured electromyography (EMG) activity of both masseters and temporalis muscles at maximum bite force using the MYOMED_932TM device. Their measurements compared baseline and 12‐month conditions.

Bernard et al. (2019) prioritized clinical and radiographic parameters including bone loss, probing depths, bleeding on probing (BoP), and plaque index. They utilized ImageJ software for radiographic measurements and conducted clinical examinations at baseline, loading, and at 1‐ and 2‐year follow‐ups. The authors presented results as mean values with standard deviations for both immediate and conventional loading groups.

Vercruyssen et al. (2016) uniquely focused on surgical accuracy and surgical procedure time, employing CT matching with Mimics software and CBCT assessment to evaluate implant placement precision between planned and actual outcomes. They measured deviations at the entry point, apex, and angular deviation, presenting data as point estimates of deviation. Results were reported as mean, median, and range values. Time points included baseline planning images compared with post‐surgery confirmatory imaging.

The included studies did not report the methods reference used to evaluate clinician satisfaction; Insertion Torque; Surgical and prosthetic complications; Occlusal force/area; EMG activity; Probing depth. The reported references for Implant stability; Bone loss; Plaque index; BoP; Accuracy of implant planning are reported in Table S5. The different ClinROs/CROMs utilized are reported in Table 4.

## 4 Discussion

This systematic review revealed several key findings regarding outcome measures in implant loading protocols for edentulous maxilla. VAS emerged as the predominant assessment tool, utilized in 80% of the included studies, primarily evaluating patient satisfaction, quality of life, pain, and functional outcomes (Bernard et al. 2019; Marković et al. 2022; Peñarrocha‐Oltra et al. 2014; Vercruyssen et al. 2016). Additionally, while other instruments like the OHIP questionnaires and McGill Pain Questionnaire were also used, the variation in assessment tools highlights a lack of standardization in reporting outcomes across different implant loading studies. A total of 12 ClinROs were identified, including implant stability, insertion torque, occlusal force/area, electromyography (EMG) activity, bone loss, probing depths, BoP, plaque index, accuracy of implant placement, surgical time, clinician esthetic satisfaction, and surgical/prosthetic complications (Marković et al. 2022; Montero et al. 2021; Bernard et al. 2019; Vercruyssen et al. 2016).

While convenient, VAS scores can be susceptible to response bias, lack comprehensive validation (Karcioglu et al. 2018), and pose challenges in interpretability compared to multi‐item validated questionnaires (Slade 1997). Validated, multi‐item instruments like the Oral Health Impact Profile (OHIP‐19, OHIP‐20) were also utilized to capture oral health‐related quality of life spanning multiple domains (Marković et al. 2022; Montero et al. 2021). Condition‐specific measures evaluated aspects such as chewing ability and masticatory performance during oral functions (Montero et al. 2021). The McGill Pain Questionnaire provided a more comprehensive assessment of pain characteristics (Vercruyssen et al. 2016).

Among the various OHIP versions, OHIP‐19 and OHIP‐20 offer distinct advantages. Derived from the original OHIP‐49 questionnaire, they are more concise, reducing participant burden and increasing feasibility for use in clinical trials or large‐scale studies (Slade and Spencer 1994). Moreover, OHIP‐19 and OHIP‐20 were specifically developed for partially dentate or edentulous populations, ensuring relevance to the domains assessed. In contrast, OHIP‐14, while highly abbreviated, was developed primarily for dentate individuals and may lack sensitivity for assessing the unique challenges faced by those with significant tooth loss or edentulism (Allen and Locker 2002; Slade 1997). By focusing on relevant domains like functional limitation, physical pain, and psychosocial disability specific to oral diseases, OHIP‐19 and OHIP‐20 provide a realistic evaluation of oral health impacts on quality of life from the patient's perspective (Allen and Locker 2002; Montero et al. 2012).

The included studies demonstrated significant inconsistency in CROMs methodology and depth of evaluation. This inconsistency reflects the absence of standardized approaches for clinician assessments in implant loading studies. Each study employed distinctly different measurement approaches without consensus on specific ClinROs, limiting direct comparisons between different loading protocols in the edentulous maxilla. Marković et al. (2022) provided the most comprehensive approach to CROMs, utilizing both objective measurements (Resonance Frequency Analysis, insertion torque) and subjective assessments (VAS for esthetic satisfaction). In addition, they were the only authors to report interrater and intrarater reliability testing. Montero et al. (2021) employed PRESCALE, EMG to evaluate functional parameters but focused primarily on mechanical outcomes rather than comprehensive clinical assessments. Bernard et al. (2019) uniquely prioritized periodontal health metrics employing clinical examination. Vercruyssen et al. (2016) adopted a more limited approach to CROMs, evaluating surgical accuracy through CT matching and reporting the mean and range of the surgical procedure duration, which can provide insights into treatment complexity and efficiency but does not directly capture clinician assessments of outcomes such as esthetic satisfaction or functional restoration.

While several studies reported PROs between immediate and delayed loading up to 1 year follow‐up (Bernard et al. 2019; Montero et al. 2021; Peñarrocha‐Oltra et al. 2014), only one study by Bernard et al. (2019) reported 2 years follow‐up after prosthesis placement. Limited follow‐up durations in some trials potentially restrict the ability to discern meaningful differences or assess the longevity of findings. Conversely, Vercruyssen et al. (2016) evaluated pain intensity, pain quality, swelling, and tolerability of the procedure daily for 7 days after implant placement. While frequent interventions can lead to increased patient discomfort, higher costs, and reduced overall satisfaction (Karimbux et al. 2023), longer‐term data with different visit intervals is needed to comprehensively evaluate PROs, ClinROs, and their potential changes over time. Nevertheless, ClinRO evaluation may need a longer period of follow‐up to assess domains like prosthetic complications, number of visits needed for maintenance, and esthetic satisfaction. In the current review, the maximum follow‐up of 2 years in the included studies is relatively short compared to the expected lifetime of implant prostheses. Further studies with different maintenance intervals and follow‐up periods are needed to define the most appropriate study settings for reporting PROMs and CROMs.

Another factor that warrants consideration is the potential influence of the mandibular dentition status on PROs following maxillary implant treatment. Several of the included studies involved patients with varying mandibular conditions, ranging from natural dentition to partial or complete edentulism with removable dentures or implant prostheses. While some studies attempted to establish balanced occlusal contacts (Bernard et al. 2019; Vercruyssen et al. 2016), the interaction between the new maxillary implant prosthesis and the existing mandibular condition may impact oral functions like chewing ability and overall satisfaction.

A critical finding of our review was the absence of detailed reporting on outcome assessor training and calibration protocols. Among the included studies, only Marković et al. (2022) reported testing interrater and intrarater reliability, while Peñarrocha‐Oltra et al. (2014) reported data collection by a trained clinician, though without elaborating on the training methodology or calibration process. This observation raises important concerns about the standardization and reliability of outcome assessments, particularly for subjective CROMs. The ID‐COSM consensus emphasizes the importance of validated measurement instruments for capturing outcomes in implant dentistry (Tonetti et al. 2023); however, the validity of these instruments depends significantly on proper assessor training and calibration. The lack of reported calibration protocols could potentially affect the reliability and comparability of findings across studies, particularly for subjective measures such as esthetic outcomes and patient satisfaction measures (Mercieca‐Bebber et al. 2016).

This systematic review has some limitations that must be considered when interpreting the findings. The included evidence base comprised a relatively small number of studies with considerable heterogeneity in terms of outcome measures, assessment strategies, and follow‐up durations. Variations in case definition of loading timelines (e.g., immediate vs. early vs. delayed) present another source of heterogeneity. It is important to acknowledge the limited geographic diversity represented in the included trials, which were predominantly conducted in European university settings. Cultural influences, racial/ethnic backgrounds, and variations in access to care or healthcare systems may impact patient expectations, experiences, and priorities regarding oral health and implant treatment.

While established tools such as OHIP‐19, OHIP‐20, and various VAS scales were utilized to evaluate patient satisfaction and oral health‐related quality of life, none of these tools were specifically validated to assess the differential impact of immediate versus early/delayed prosthetic delivery timing on PROs. As highlighted by Needleman et al. (2023), the use of PROMs could be for generic or condition‐specific assessment. The tools employed across the included studies were primarily validated for general assessment of prosthetic outcomes in edentulous patients but lack specific validation for detecting differences based on restoration timing protocols. Future research should focus on developing and validating PROMs that can more sensitively detect nuanced differences in PROs related specifically to the timing of prosthetic delivery after implant placement.

A notable methodological consideration in our review was the exclusion of single‐arm and retrospective studies. While this decision limited the number of studies included, it enhanced the quality and reliability of our findings by focusing on prospective comparative evidence. Although our systematic review initially planned to present outcomes and measures separately (PROs, ClinROs, PROMs, and CROMs) as mentioned in the focused questions, the heterogeneous nature of the reported data necessitated an integrated approach. This resulted in presenting outcomes alongside their respective measures to provide a clearer synthesis of the available evidence.

One of the notable strengths of this systematic review lies in its comprehensive and rigorous methodology. The detailed inspection of the PROMs and CROMs used for outcome measurement and implementation strategies revealed important patterns like diverse assessment intervals ranging from daily evaluations over 1 week (Vercruyssen et al. 2016) to extended follow‐ups of up to 2 years (Bernard et al. 2019), identification of varying baseline measurement points (prior to implant placement, pretreatment, posttreatment), comprehensive analysis of assessment tools' implementation (including OHIP‐19/20 for quality of life domains, VAS for multiple outcomes, and McGill Pain Questionnaire metrics), and critical evaluation of clinician calibration protocols.

Furthermore, the authors employed well‐established risk of bias assessment tools, such as the Cochrane RoB 2 and ROBINS‐I, to critically evaluate the methodological quality of the included studies. The risk of bias assessment revealed that most RCTs demonstrated some concerns across various domains, while the single non‐randomized trial showed moderate risk of bias. However, given the descriptive nature of our review and the substantial heterogeneity in outcome measures across studies, we did not weight studies based on risk of bias. Instead, these assessments serve to contextualize the quality of available evidence and inform recommendations for future research methodology.

The systematic review highlights the need for standardization and harmonization of outcome measures in this field, which could facilitate more robust evidence synthesis and cross‐study comparisons. By recognizing the paucity of comprehensive CROMs data, the authors emphasize the importance of developing and incorporating validated clinician‐reported evaluations in future research, thereby promoting a more holistic interpretation of treatment effectiveness from both patient and clinician perspectives.

## 5 Conclusion

PROs were predominantly assessed using VAS to evaluate patient satisfaction, pain, function, and esthetics followed by OHIP Questionnaire for quality‐of‐life assessment in immediate versus early/delayed loading protocols. ClinROs showed no standardized approach, creating significant heterogeneity in the reported outcomes. Standardization of assessment methods and reporting of PROMs and CROMs is needed to optimize analyzing patient‐centered outcomes.

## Author Contributions

**Claudio Mendes Pannuti:** conceptualization, writing – review and editing, writing – original draft, project administration, supervision. **Mohamed A. Hassan:** investigation, writing – original draft, writing – review and editing, methodology, software, formal analysis, data curation. **Isabella Neme Ribeiro Reis:** writing – review and editing, writing – original draft, investigation, formal analysis, software, methodology. **Cristina Cunha Villar:** writing – review and editing, writing – original draft, methodology. **Helena Francisco:** writing – original draft, methodology, conceptualization, supervision. **Giuseppe A. Romito:** writing – review and editing, supervision, methodology, conceptualization, project administration.

## Conflicts of Interest

The authors declare no conflicts of interest.

## Supporting information


**Table S1.** PRISMA checklist.

**Table S2.** Search strategies for each database.

**Table S3.** List of the excluded studies (*N* = 63).

**Table S4.** PROs/PROMs references and validation data.

**Table S5.** CROs and CROMs references.

## Data Availability

The data that support the findings of this study are available from the corresponding author upon reasonable request.
