# Supplementary material for: Patient‐ and Clinician‐Reported Outcomes and Outcome Measures Evaluating Timing of Implant Loading in the Edentulous Maxilla: A Systematic Review of Prospective Studies
Source: Clin Oral Implants Res. 2026 Feb 24;37(Suppl 30):S332–45. doi: 10.1111/clr.14451 (PMC12930122; doi:10.1111/clr.14451)
Supplement: Supplementary file 3 — Table S3. List of the excluded studies (N = 63). [file CLR-37-S332-s002.docx]

Table S3 – List of the excluded studies (N = 63)

| Study | Reason for exclusion | Subgroup |
| --- | --- | --- |
| (Tang et al. 2018) | Not in English | - |
| (Zhao et al. 2014) | Not in English | - |
| (Pineau et al. 2018) | Not in English / Zygomatic | Zygomatic implant |
| (Nagib et al. 2024) | Zygomatic implant | Zygomatic implant |
| (Agliardi et al. 2021) | Zygomatic implant | Zygomatic implant |
| (Fernández-Ruiz et al. 2021) | Zygomatic implant | Zygomatic implant |
| (Davó et al. 2018) | Zygomatic implant | Zygomatic implant |
| (Agliardi et al. 2017) | Zygomatic implant | Zygomatic implant |
| (Atalay et al. 2017) | Zygomatic implant | Zygomatic implant |
| (Davó and Pons 2015) | Zygomatic implant | Zygomatic implant |
| (Aparicio et al. 2014) | Zygomatic implant | Zygomatic implant |
| (Aboelez et al. 2024) | Reporting patient satisfaction for Vertical versus angled immediately loaded implants | Overdenture/ Prosthetic components |
| (Xie et al. 2024) | Does not evaluate PROMS or ClinROs | - |
| (Bouhy et al. 2023) | Maxillary implant overdenture retained by four unsplinted attachments and opposed by a natural or fixed dentition | Overdenture/ Prosthetic components |
| (Choudhary et al. 2023) | Less than 10 cases | - |
| (Komagamine et al. 2023) | Does not report different loading protocols. | - |
| (Onclin et al. 2023) | Reporting PROMS for implant overdentures retained with bars or solitary attachments. | Overdenture/ Prosthetic components |
| (Pomares-Puig, Sánchez-Garcés, and Jorba-García 2023) | Reporting PROMs and patient quality of life between Dynamic and static computer-assisted implant surgery. | Implant Placement |
| (Slot et al. 2023) | Reporting patient satisfaction for maxillary overdentures supported by four or six implants- all implants. | Number of implants |
| (Agustín-Panadero et al. 2022) | Reporting patient satisfaction for Implant-supported overdenture with horizontal insertion. | Overdenture/ Prosthetic components |
| (Caramês et al. 2022) | Does not evaluate loading protocols. | - |
| (Fayek et al. 2022) | Primary outcome is different attachments for mandibular overdenture. | Overdenture/ Prosthetic components |
| (Mo, Hjortsjö, and Jokstad 2022) | Reporting patient satisfaction for removable prosthesis retained by three implants fitted with low- profile stud attachment. | Overdenture/ Prosthetic components |
| (Onclin et al. 2022) | Reporting PROMs for maxillary overdenture with palatal coverage and solitary attachments. | Overdenture/ Prosthetic components |
| (Slot et al. 2022) | Reporting PROMS for Maxillary bar overdentures on four or six posterior implants. | Number of implants |
| (Van Den Borre et al. 2022) | PROMS and ClinROs for the additively manufactured sub-periosteal jaw. | Implant placement |
| (Fonteyne et al. 2021) | Speech‐ and oral health‐related quality of life in patients with implant‐supported overdentures. | Overdenture/ Prosthetic components |
| (Kilic et al. 2021) | Comparing complete denture and implant supported over denture | - |
| (Van Doorne et al. 2021) | Report PROMs for maxillary mini dental implant overdentures (all with delayed loading, single time point | Overdenture/ Prosthetic components |
| (Bolding and Reebye 2020) | Less than 10 participants | - |
| (Bouhy et al. 2020) | Reporting Patient-reported outcome measures (PROMs) of maxillary removable prostheses retained by 4 implant-supported – unsplinted attachments | Overdenture/ Prosthetic components |
| (Ferrer et al. 2020) | Comparing two different types of implant overdenture attachments. | Overdenture/ Prosthetic components |
| (Kusumoto et al. 2020) | Implants were not placed during the study | - |
| (Mahanna et al. 2020) | Reports OHQoL with different connectors for overdenture | Overdenture/ Prosthetic components |
| (Van Doorne et al. 2020) | Flapless mini-implants retained over denture | Implant placement |
| (Fonteyne et al. 2019) | Cohort of mini-implant overdenture | Implant placement |
| (Nicolau et al. 2019) | Partially edentulous | - |
| (Park, Shin, and Lee 2019) | Reporting proms for Bar versus ball attachments for maxillary four‐implant retained overdentures | Overdenture/ Prosthetic components |
| (Slot et al. 2019) | Compares four bar- and six bar-connected implants | Overdenture/ Prosthetic components |
| (Zembic et al. 2019) | Evaluates implant-retained overdentures (IODs) on two implants | Overdenture/ Prosthetic components |
| (Johar 2018) | Different types of prosthesis, maxilla, and mandible | - |
| (Boven et al. 2017) | All with delayed implants placement | Implant placement |
| (Erkapers et al. 2017) | Single group with immediate implants (single time point) | Implant placement |
| (Li et al. 2017) | Data regarding the method used to measure patient satisfaction were not reported | - |
| (Meloni et al. 2017) | Did not report proms | - |
| (Esposito et al. 2018) | Zygomatic implant | Zygomatic implant |
| (Busenlechner et al. 2016) | Did not report proms | - |
| (Fürhauser et al. 2016) | Single group with immediate implant placement | Implant placement |
| (Heuberer et al. 2016) | Less than 10 participants | - |
| (Pozzi, Tallarico, and Moy 2016) | Evaluate the clinical performance of a 4-implant overdenture | Overdenture/ Prosthetic components |
| (Slot et al. 2016) | Four and six-implant maxillary overdenture | Number of implants |
| (Zhang et al. 2016) | Delayed implant placement | Implant placement |
| (Browaeys et al. 2015) | Does not report PROMs | - |
| (Kuoppala and Raustia 2015) | Evaluate the outcome of maxillary implant overdenture treatment | Overdenture/ Prosthetic components |
| (Misumi et al. 2015) | Single group with immediate implant placement | Implant placement |
| (Mundt et al. 2015) | Complete dentures with mini-implants | Overdenture/ Prosthetic components |
| (Wu et al. 2015) | Zygomatic implants | Zygomatic implant |
| (Yamada et al. 2015) | Measure VAS for pain with immediate implants. | Implant placement |
| (Agliardi et al. 2014) | First published in 2012 | - |
| (Pera et al. 2014) | Does not report PROMs | - |
| (Slot, Raghoebar, Vissink, and Henny J.A. Meijer 2014) | Patient satisfaction with different numbering of implant. | Number of implants |
| (Slot, Raghoebar, Vissink, and Henny J. A. Meijer 2014) | Different numbering of implant | Number of implants |
| (Vercruyssen et al. 2014) | The study aimed to compare guided and conventional surgery for implant placement | Surgical technique |

References

Aboelez, Marwa Ahmed, Christine Raouf Micheal Ibrahim, Mohamad Hossam El-Din Helmy, and Moustafa Abdou Elsyad. 2024. “Vertical versus Angled Immediately Loaded Implants for Assisting Maxillary Overdentures with Locator Attachments: A Preliminary Results of One-Year Randomized Clinical Trial.” *Clinical Implant Dentistry and Related Research* 26(1):103–12. doi: 10.1111/cid.13291.

Agliardi, E. L., D. Romeo, S. Panigatti, M. de Araújo Nobre, and P. Maló. 2017. “Immediate Full-Arch Rehabilitation of the Severely Atrophic Maxilla Supported by Zygomatic Implants: A Prospective Clinical Study with Minimum Follow-up of 6 Years.” *International Journal of Oral and Maxillofacial Surgery* 46(12):1592–99. doi: 10.1016/j.ijom.2017.05.023.

Agliardi, Enrico L., Alessandro Pozzi, Christian F. J. Stappert, Riccardo Benzi, Davide Romeo, and Enrico Gherlone. 2014. “Immediate Fixed Rehabilitation of the Edentulous Maxilla: A Prospective Clinical and Radiological Study after 3 Years of Loading.” *Clinical Implant Dentistry and Related Research* 16(2):292–302. doi: 10.1111/j.1708-8208.2012.00482.x.

Agliardi, Enrico Luigi, Stefano Panigatti, Davide Romeo, Lavinia Sacchi, and Enrico Gherlone. 2021. “Clinical Outcomes and Biological and Mechanical Complications of Immediate Fixed Prostheses Supported by Zygomatic Implants: A Retrospective Analysis from a Prospective Clinical Study with up to 11 Years of Follow-Up.” *Clinical Implant Dentistry and Related Research* 23(4):612–24. doi: 10.1111/cid.13017.

Agustín-Panadero, Rubén, Jose Félix Mañes-Ferrer, Naia Bustamante-Hernández, María Fernanda Solá-Ruíz, Carla Fons-Badal, and Lucía Fernández-Estevan. 2022. “Implant-Supported Overdenture with Horizontal Insertion for Treating the Edentulous Atrophic Maxilla: A Case Series.” *The Journal of Prosthetic Dentistry* 128(5):942–48. doi: 10.1016/j.prosdent.2021.02.008.

Aparicio, Carlos, Carolina Manresa, Karen Francisco, Arnau Aparicio, Jonas Nunes, Pedro Claros, and Josep M. Potau. 2014. “Zygomatic Implants Placed Using the Zygomatic Anatomy-Guided Approach versus the Classical Technique: A Proposed System to Report Rhinosinusitis Diagnosis.” *Clinical Implant Dentistry and Related Research* 16(5):627–42. doi: 10.1111/cid.12047.

Atalay, Belir, Özge Doganay, Banu Karayazgan Saraçoglu, Özgür Bultan, and Günter Hafiz. 2017. “Clinical Evaluation of Zygomatic Implant-Supported Fixed and Removable Prosthesis.” *Journal of Craniofacial Surgery* 28(1):185. doi: 10.1097/SCS.0000000000003204.

Bolding, S. L., and U. N. Reebye. 2020. “Robotic-Guided Dental Implant Placement in Fully Edentulous Patients: Preliminary Results of a Prospective Multi-Center Clinical Study.” *Journal of Oral and Maxillofacial Surgery* 78(10):e22–23. doi: 10.1016/j.joms.2020.07.064.

Bouhy, Alice, Marc Lamy, Yaman Altaep, and France Lambert. 2023. “Maxillary Implant Overdenture Retained by Four Unsplinted Attachments and Opposed by a Natural or Fixed Dentition: Five‐year Clinical Outcomes. A Prospective Case Series.” *Clinical Oral Implants Research* 34(4):285–96. doi: 10.1111/clr.14033.

Bouhy, Alice, Eric Rompen, Marc Lamy, Caroline Legros, Geoffrey Lecloux, and France Lambert. 2020. “Maxillary Implant Overdenture Retained by Four Unsplinted Attachments and Opposed by a Natural or Fixed Dentition: One-Year Clinical Outcomes.” *Clinical Oral Implants Research* 31(8):747–67. doi: 10.1111/clr.13623.

Boven, G. C., J. W. A. Slot, G. M. Raghoebar, A. Vissink, and H. J. A. Meijer. 2017. “Maxillary Implant‐supported Overdentures Opposed by (Partial) Natural Dentitions: A 5‐year Prospective Case Series Study.” *Journal of Oral Rehabilitation* 44(12):988–95. doi: 10.1111/joor.12557.

Browaeys, Hilde, Melissa Dierens, Christian Ruyffelaert, Carine Matthijs, Hugo De Bruyn, and Stefan Vandeweghe. 2015. “Ongoing Crestal Bone Loss around Implants Subjected to Computer‐Guided Flapless Surgery and Immediate Loading Using the All‐on‐4® Concept.” *Clinical Implant Dentistry and Related Research* 17(5):831–43. doi: 10.1111/cid.12197.

Busenlechner, Dieter, Georg Mailath-Pokorny, Robert Haas, Rudolf Fürhauser, Carina Eder, Bernhard Pommer, and Georg Watzek. 2016. “Graftless Full-Arch Implant Rehabilitation with Interantral Implants and Immediate or Delayed Loading—Part II: Transition from the Failing Maxillary Dentition.” *The International Journal of Oral & Maxillofacial Implants* 1150–55. doi: 10.11607/jomi.4326.

Caramês, João Manuel Mendez, Filipe Araújo Vieira, Gonçalo Bártolo Caramês, Ana Catarina Pinto, Helena Cristina Oliveira Francisco, and Duarte Nuno Da Silva Marques. 2022. “Guided Bone Regeneration in the Edentulous Atrophic Maxilla Using Deproteinized Bovine Bone Mineral (DBBM) Combined with Platelet-Rich Fibrin (PRF)—A Prospective Study.” *Journal of Clinical Medicine* 11(3):894. doi: 10.3390/jcm11030894.

Choudhary, Ashish, Shairy Vashist, Megha Chopra, and Aparana Sharma. 2023. “A Fixed Reconstruction and Immediate Rehabilitation of Fully Edentulous Arch Using the All-on-Four Concept: Case Series.” *Journal of Long-Term Effects of Medical Implants* 33(2). doi: 10.1615/JLongTermEffMedImplants.2022044515.

Davó, Rubén, Pietro Felice, Roberto Pistilli, Carlo Barausse, Carlos Marti-Pages, Ada Ferrer-Fuertes, Daniela Rita Ippolito, and Marco Esposito. 2018. “Immediately Loaded Zygomatic Implants vs Conventional Dental Implants in Augmented Atrophic Maxillae: 1-Year Post-Loading Results from a Multicentre Randomised Controlled Trial.” *European Journal of Oral Implantology* 11(2):145–61.

Davó, Rubén, and Olivia Pons. 2015. “5-Year Outcome of Cross-Arch Prostheses Supported by Four Immediately Loaded Zygomatic Implants: A Prospective Case Series.” *European Journal of Oral Implantology* 8(2):169–74.

Erkapers, Maria, Susanna Segerström, Karl Ekstrand, Russell A. Baer, Joseph A. Toljanic, and Andreas Thor. 2017. “The Influence of Immediately Loaded Implant Treatment in the Atrophic Edentulous Maxilla on Oral Health Related Quality of Life of Edentulous Patients: 3-Year Results of a Prospective Study.” *Head & Face Medicine* 13(1):21. doi: 10.1186/s13005-017-0154-0.

Esposito, Marco, Rubén Davó, Carlos Marti-Pages, Ada Ferrer-Fuertes, Carlo Barausse, Roberto Pistilli, Daniela Rita Ippolito, and Pietro Felice. 2018. “Immediately Loaded Zygomatic Implants vs Conventional Dental Implants in Augmented Atrophic Maxillae: 4 Months Post-Loading Results from a Multicentre Randomised Controlled Trial.” *European Journal of Oral Implantology* 11(1):11–28.

Fayek, Nancy, Ahmed Mahrous, Ahmed Shaaban, and Moustafa ELsyad. 2022. “Patient Satisfaction and Prosthetic Complications of Maxillary Implant Overdentures Opposing Mandibular Implant Overdentures with Bar, Telescopic, and Stud Attachments: A 1-Year Prospective Trial.” *The International Journal of Oral & Maxillofacial Implants* 37(5):1044–54. doi: 10.11607/jomi.9610.

Fernández-Ruiz, Juan Alberto, Mariano Sánchez-Siles, Yolanda Guerrero-Sánchez, Jesús Pato-Mourelo, and Fabio Camacho-Alonso. 2021. “Evaluation of Quality of Life and Satisfaction in Patients with Fixed Prostheses on Zygomatic Implants Compared with the All-on-Four Concept: A Prospective Randomized Clinical Study.” *International Journal of Environmental Research and Public Health* 18(7):3426. doi: 10.3390/ijerph18073426.

Ferrer, José Félix, Lucía Fernández-Estevan, Eduardo Selva-Otaolaurruchi, Carlos Labaig-Rueda, María Fernanda Solá-Ruíz, and Rubén Agustín-Panadero. 2020. “Maxillary Implant-Supported Overdentures: Mechanical Behavior Comparing Individual Axial and Bar Retention Systems. A Cohort Study of Edentulous Patients.” *Medicina* 56(3):139. doi: 10.3390/medicina56030139.

Fonteyne, Ester, Eline Burms, Carine Matthys, Kristiane Van Lierde, and Hugo De Bruyn. 2021. “Four‐implant‐supported Overdenture Treatment in the Maxilla. Part II : Speech‐ and Oral Health‐related Quality of Life in Patients with Implant‐supported Overdentures in the Maxilla—A Prospective 3‐year Follow‐up.” *Clinical Implant Dentistry and Related Research* 23(5):680–91. doi: 10.1111/cid.13034.

Fonteyne, Ester, Luc Van Doorne, Laurence Becue, Carine Matthys, Ewald Bronckhorst, and Hugo De Bruyn. 2019. “Speech Evaluation during Maxillary Mini‐dental Implant Overdenture Treatment: A Prospective Study.” *Journal of Oral Rehabilitation* 46(12):1151–60. doi: 10.1111/joor.12852.

Fürhauser, Rudolf, Georg Mailath-Pokorny, Robert Haas, Dieter Busenlechner, Georg Watzek, and Bernhard Pommer. 2016. “Patient-Perceived Morbidity and Subjective Functional Impairment Following Immediate Transition from a Failing Dentition to Fixed Implant Rehabilitation.” *The International Journal of Oral & Maxillofacial Implants* 651–56. doi: 10.11607/jomi.4471.

Heuberer, Simone, Christian Ulm, Konstantin Zauza, Werner Zechner, Georg Watzek, and Gabriella Dvorak. 2016. “Effectiveness of Subperiosteal Bone Anchor (Onplant) Placement in the Anterior Highly Atrophic Maxilla for Cross-Arch Prosthetic Rehabilitation: Results from a Pilot Study.” *European Journal of Oral Implantology* 9(3):291–97.

Johar, Aiman O. 2018. “Clinical Performance of Implant Overdenture Versus Fixed Detachable Prosthesis.” *The Journal of Contemporary Dental Practice* 19(12):1481–87. doi: 10.5005/jp-journals-10024-2453.

Kilic, Kerem, Bahar Sayin, Firuzan Ozer, and Sibel Akin. 2021. “Influence of Conventional Complete Dentures and Different Attachment Types in Implant-Supported Overdentures on Quality of Life and Nutritional Status in Edentulous Geriatric Patients.” *The International Journal of Prosthodontics* 7–12. doi: 10.11607/ijp.6690.

Komagamine, Yuriko, Manabu Kanazawa, Anna Miyayasu, Yoko Uehara, Masataka Watanabe, Namano Sahaprom, Trang Bui Ngoc Huyen, Maiko Iwaki, Daisuke Sato, and Shunsuke Minakuchi. 2023. “The Effect of Single-Implant Overdentures on Cognitive Function in Older Adults: A 3-Year Follow-up Report.” *Journal of Dentistry* 136:104632. doi: 10.1016/j.jdent.2023.104632.

Kuoppala, Ritva, and Aune Raustia. 2015. “Preliminary Observations Regarding Treatment Outcomes in Patients Treated with Maxillary Implant Overdentures in a University Clinic.” *The International Journal of Prosthodontics* 28(6):637–40. doi: 10.11607/ijp.4384.

Kusumoto, Yuriko, Jyoji Tanaka, Keizo Miyoshi, Daisuke Higuchi, Yoko Sato, and Kazuyoshi Baba. 2020. “Impact of Implant Superstructure Type on Oral Health-Related Quality of Life in Edentulous Patients.” *Clinical Implant Dentistry and Related Research* 22(3):319–24. doi: 10.1111/cid.12895.

Li, Sha, Ping Di, Yu Zhang, and Ye Lin. 2017. “Immediate Implant and Rehabilitation Based on All-on-4 Concept in Patients with Generalized Aggressive Periodontitis: A Medium-Term Prospective Study.” *Clinical Implant Dentistry and Related Research* 19(3):559–71. doi: 10.1111/cid.12483.

Mahanna, Fatma, Moustafa Elsyad, Samah Mourad, and Heba Abozaed. 2020. “Satisfaction and Oral Health–Related Quality of Life of Different Attachments Used for Implant-Retained Overdentures in Subjects with Resorbed Mandibles: A Crossover Trial.” *The International Journal of Oral & Maxillofacial Implants* 35(2):423–31. doi: 10.11607/jomi.7869.

Meloni, Silvio Mario, Marco Tallarico, Milena Pisano, Erta Xhanari, and Luigi Canullo. 2017. “Immediate Loading of Fixed Complete Denture Prosthesis Supported by 4–8 Implants Placed Using Guided Surgery: A 5-Year Prospective Study on 66 Patients with 356 Implants.” *Clinical Implant Dentistry and Related Research* 19(1):195–206. doi: 10.1111/cid.12449.

Misumi, Saori, Tetsuji Nakamoto, Yusuke Kondo, Taro Mukaibo, Chihiro Masaki, and Ryuji Hosokawa. 2015. “A Prospective Study of Changes in Oral Health-Related Quality of Life during Immediate Function Implant Procedures for Edentulous Individuals.” *Clinical Oral Implants Research* 26(6):696–700. doi: 10.1111/clr.12371.

Mo, Arild, Carl Hjortsjö, and Asbjørn Jokstad. 2022. “Maxillary Overdenture on Three Implants Retained by Low‐profile Stud Attachments – A Prospective Cohort Study.” *Journal of Oral Rehabilitation* 49(11):1069–79. doi: 10.1111/joor.13364.

Mundt, Torsten, Christian Schwahn, Thomas Stark, and Reiner Biffar. 2015. “Clinical Response of Edentulous People Treated with Mini Dental Implants in Nine Dental Practices.” *Gerodontology* 32(3):179–87. doi: 10.1111/ger.12066.

Nagib, Mohamed A., Fakhreldin Hassan Abdel-Rahman, Salah A. Hegazy, Abdullah M. Ibrahim, and Ahmed Habib. 2024. “Evaluation of Quality of Life and Satisfaction with Fixed Prostheses on Zygomatic Implants vs All-on-Four Concept: A Randomized Clinical Study.” *The Journal of Contemporary Dental Practice* 25(2):141–47. doi: 10.5005/jp-journals-10024-3632.

Nicolau, P., Guerra, R. Reis, T. Krafft, K. Benz, and Jackowski. 2019. “10-Year Outcomes with Immediate and Early Loaded Implants with a Chemically Modified SLA Surface.” *Quintessence International* 50(2):114–24. doi: 10.3290/j.qi.a41664.

Onclin, Pieter, G. Carina Boven, Arjan Vissink, Henny Ja Meijer, and Gerry M. Raghoebar. 2023. “Maxillary Implant Overdentures Retained with Bars or Solitary Attachments: A 5-Year Randomised Controlled Trial.” *Journal of Prosthodontic Research* 67(3):400–409. doi: 10.2186/jpr.JPR_D_22_00076.

Onclin, Pieter, Caroline M. Speksnijder, Henny J. A. Meijer, Arjan Vissink, and Gerry M. Raghoebar. 2022. “The Performance of Two-Implant Overdentures in the Atrophic Maxilla: A Case Series with 1-Year Follow-Up.” *International Journal of Implant Dentistry* 8(1):64. doi: 10.1186/s40729-022-00460-0.

Park, Jin‐Hong, Sang‐Wan Shin, and Jeong‐Yol Lee. 2019. “Bar versus Ball Attachments for Maxillary Four‐implant Retained Overdentures: A Randomized Controlled Trial.” *Clinical Oral Implants Research* 30(11):1076–84. doi: 10.1111/clr.13521.

Pera, Paolo, Maria Menini, Marco Bevilacqua, Paolo Pesce, Francesco Pera, Alessio Signori, and Tiziano Tealdo. 2014. “Factors Affecting the Outcome in the Immediate Loading Rehabilitation of the Maxilla: A 6-Year Prospective Study.” *The International Journal of Periodontics & Restorative Dentistry* 34(5):657–65. doi: 10.11607/prd.1970.

Pineau, Mallouel, Romain Nicot, Ludovic Lauwers, Joël Ferri, and Gwénaël Raoul. 2018. “[Zygomatic implants in our daily practice. Part II: Prosthetic rehabilitation and effect on quality of life].” *Swiss Dental Journal* 128(9):694–700.

Pomares-Puig, Carmen, M. Angeles Sánchez-Garcés, and Adrià Jorba-García. 2023. “Dynamic and Static Computer-Assisted Implant Surgery for Completely Edentulous Patients. A Proof of a Concept.” *Journal of Dentistry* 130:104443. doi: 10.1016/j.jdent.2023.104443.

Pozzi, Alessandro, Marco Tallarico, and Peter K. Moy. 2016. “Four-Implant Overdenture Fully Supported by a CAD-CAM Titanium Bar: A Single-Cohort Prospective 1-Year Preliminary Study.” *The Journal of Prosthetic Dentistry* 116(4):516–23. doi: 10.1016/j.prosdent.2016.03.015.

Slot, Wim, Gerry M. Raghoebar, Marco S. Cune, Arjan Vissink, and Henny J. A. Meijer. 2016. “Maxillary Overdentures Supported by Four or Six Implants in the Anterior Region: 5‐year Results from a Randomized Controlled Trial.” *Journal of Clinical Periodontology* 43(12):1180–87. doi: 10.1111/jcpe.12625.

Slot, Wim, Gerry M. Raghoebar, Marco S. Cune, Arjan Vissink, and Henny J. A. Meijer. 2019. “Four or Six Implants in the Maxillary Posterior Region to Support an Overdenture: 5‐year Results from a Randomized Controlled Trial.” *Clinical Oral Implants Research* 30(2):169–77. doi: 10.1111/clr.13403.

Slot, Wim, Gerry M. Raghoebar, Marco S. Cune, Arjan Vissink, and Henny J. A. Meijer. 2022. “Maxillary Bar Overdentures on Four or Six Posterior Implants: 10‐year Results from a Randomized Clinical Trial.” *Clinical Oral Implants Research* 33(11):1147–56. doi: 10.1111/clr.13997.

Slot, Wim, Gerry M. Raghoebar, Marco S. Cune, Arjan Vissink, and Henny J. A. Meijer. 2023. “Maxillary Overdentures Supported by Four or Six Implants in the Anterior Region: 10‐year Randomized Controlled Trial Results.” *Journal of Clinical Periodontology* 50(1):36–44. doi: 10.1111/jcpe.13726.

Slot, Wim, Gerry M. Raghoebar, Arjan Vissink, and Henny J.A. Meijer. 2014. “A Comparison between 4 and 6 Implants in the Maxillary Posterior Region to Support an Overdenture; 1‐year Results from a Randomized Controlled Trial.” *Clinical Oral Implants Research* 25(5):560–66. doi: 10.1111/clr.12118.

Slot, Wim, Gerry M. Raghoebar, Arjan Vissink, and Henny J. A. Meijer. 2014. “Maxillary Overdentures Supported by Anteriorly or Posteriorly Placed Implants Opposed by a Natural Dentition in the Mandible: A 1‐Year Prospective Case Series Study.” *Clinical Implant Dentistry and Related Research* 16(1):51–61. doi: 10.1111/j.1708-8208.2012.00459.x.

Tang, Ting, Lei Zhang, Yong-Fu Hou, Ying-Chun Jiang, Xiang-Dong Wang, and Ning Chen. 2018. “[A prospective study of changes in oral health-related quality of life during ‘all-on-four’ immediate restoration for edentulous individuals].” *Shanghai Kou Qiang Yi Xue = Shanghai Journal of Stomatology* 27(1):52–55.

Van Den Borre, C., M. Rinaldi, B. De Neef, N. A. J. Loomans, E. Nout, L. Van Doorne, I. Naert, C. Politis, H. Schouten, G. Klomp, L. Beckers, M. M. Freilich, and M. Y. Mommaerts. 2022. “Patient- and Clinician-Reported Outcomes for the Additively Manufactured Sub-Periosteal Jaw Implant (AMSJI) in the Maxilla: A Prospective Multicentre One-Year Follow-up Study.” *International Journal of Oral and Maxillofacial Surgery* 51(2):243–50. doi: 10.1016/j.ijom.2021.05.015.

Van Doorne, L., L. De Kock, A. De Moor, R. Shtino, E. Bronkhorst, G. Meijer, and H. De Bruyn. 2020. “Flaplessly Placed 2.4-Mm Mini-Implants for Maxillary Overdentures: A Prospective Multicentre Clinical Cohort Study.” *International Journal of Oral and Maxillofacial Surgery* 49(3):384–91. doi: 10.1016/j.ijom.2019.08.015.

Van Doorne, Luc, Ester Fonteyne, Carine Matthys, Ewald Bronkhorst, Gert Meijer, and Hugo De Bruyn. 2021. “‘Longitudinal Oral Health-Related Quality of Life in Maxillary Mini Dental Implant Overdentures after 3 Years in Function.’” *Clinical Oral Implants Research* 32(1):23–36. doi: 10.1111/clr.13677.

Vercruyssen, Marjolein, Gerlinde van de Wiele, Wim Teughels, Ignace Naert, Reinhilde Jacobs, and Marc Quirynen. 2014. “Implant- and Patient-Centred Outcomes of Guided Surgery, a 1-Year Follow-up: An RCT Comparing Guided Surgery with Conventional Implant Placement.” *Journal of Clinical Periodontology* 41(12):1154–60. doi: 10.1111/jcpe.12305.

Wu, Yiqun, Xu Dong Wang, Feng Wang, Wei Huang, Zhiyong Zhang, Zhiyuan Zhang, Darnell Kaigler, and Duohong Zou. 2015. “Restoration of Oral Function for Adult Edentulous Patients with Ectodermal Dysplasia: A Prospective Preliminary Clinical Study.” *Clinical Implant Dentistry and Related Research* 17(S2). doi: 10.1111/cid.12296.

Xie, Rui, Yuchen Liu, Hongbo Wei, Tingmin Zhang, Shizhu Bai, and Yimin Zhao. 2024. “Clinical Evaluation of Autonomous Robotic-Assisted Full-Arch Implant Surgery: A 1-Year Prospective Clinical Study.” *Clinical Oral Implants Research* 35(4):443–53. doi: 10.1111/clr.14243.

Yamada, Junichi, Hidehiro Kori, Yoshihiro Tsukiyama, Yasuyuki Matsushita, Makoto Kamo, and Kiyoshi Koyano. 2015. “Immediate Loading of Complete-Arch Fixed Prostheses for Edentulous Maxillae After Flapless Guided Implant Placement: A 1-Year Prospective Clinical Study.” *The International Journal of Oral & Maxillofacial Implants* 30(1):184–93. doi: 10.11607/jomi.3679.

Zembic, Anja, Ali Tahmaseb, Ronald Jung, Daniel Wiedemeier, and Daniel Wismeijer. 2019. “Patient-Reported Outcomes of Maxillary Edentulous Patients Wearing Overdentures Retained by Two Implants from Insertion to 4 Years.” *The International Journal of Oral & Maxillofacial Implants* 34(2):481–88. doi: 10.11607/jomi.6980.

Zhang, Xiao-Xiao, Jun-Yu Shi, Ying-Xin Gu, and Hong-Chang Lai. 2016. “Long-Term Outcomes of Early Loading of Straumann Implant-Supported Fixed Segmented Bridgeworks in Edentulous Maxillae: A 10-Year Prospective Study.” *Clinical Implant Dentistry and Related Research* 18(6):1227–37. doi: 10.1111/cid.12420.

Zhao, Xu, Ping Di, Ye Lin, Jian-hui Li, Li-xin Qiu, Jia Luo, and Hong-yan Cui. 2014. “[Implanting the edentulous jaws with ‘All-on-4’ immediate reconstruction: a preliminary clinical observation].” *Beijing Da Xue Xue Bao. Yi Xue Ban = Journal of Peking University. Health Sciences* 46(5):720–26.
