# Supplementary material for: Patient‐ and Clinician‐Reported Outcomes and Outcome Measures Evaluating Timing of Implant Loading in the Edentulous Maxilla: A Systematic Review of Prospective Studies
Source: Clin Oral Implants Res. 2026 Feb 24;37(Suppl 30):S332–45. doi: 10.1111/clr.14451 (PMC12930122; doi:10.1111/clr.14451)
Supplement: Supplementary file 4 — Table S4. PROs/PROMs references and validation data. [file CLR-37-S332-s005.docx]

Table S4 – PROs/ PROMs references and validation data

| **Study** | **PROMs** | **PROMs References** | **PROMs General validation** | **PROMs Validation in the context of implant prosthetic evaulation** |
| --- | --- | --- | --- | --- |
| **Marković et al. (2022)** | ·  OHIP-19 | Allen, Finbarr, and David Locker. 2002. “A Modified Short Version of the Oral Health Impact Profile for Assessing Health-Related Quality of Life in Edentulous Adults.” The International Journal of Prosthodontics 15(5):446–50. | **Yes** | **Yes** |
|  | ·   VAS | Miller, M. D., and D. G. Ferris. 1993. “Measurement of Subjective Phenomena in Primary Care Research: The Visual Analogue Scale.” Family Practice Research Journal 13(1):15–24. | **No** | **No** |
| **Montero et al. (2021)** | ·   OHIP-20 | Montero, Javier, Carla Macedo, Antonio López-Valverde, and Manuel Bravo. 2012. “Validation of the Oral Health Impact Profile (OHIP-20sp) for Spanish Edentulous Patients.” Medicina Oral, Patologia Oral Y Cirugia Bucal 17(3):e469-476. doi: 10.4317/medoral.17498. | **Yes** | **Yes** |
|  | ·  Chewing Ability: | Leake, J. L. 1990. “An Index of Chewing Ability.” Journal of Public Health Dentistry 50(4):262–67. doi: 10.1111/j.1752-7325.1990.tb02133.x. | **No** | **No** |
|  | ·  Self-reported oral satisfaction | Montero, J., M. Bravo, and A. Albaladejo. 2008. “Validation of Two Complementary Oral-Health Related Quality of Life Indicators (OIDP and OSS 0-10 ) in Two Qualitatively Distinct Samples of the Spanish Population.” Health and Quality of Life Outcomes 6:101. doi: 10.1186/1477-7525-6-101. | **Yes** | **No** |
|  | ·   Masticatory performance | Montero, Javier, Luis Leiva, Inmaculada Martín-Quintero, and Rocío Barrios-Rodríguez. 2021. “Chewing Performance Calculator: An Interactive Clinical Method for Quantifying Masticatory Performance.” The Journal of Prosthetic Dentistry 125(1):82–88. doi: 10.1016/j.prosdent.2019.10.006. | **Yes** | **No** |
| **Bernard et al. (2019)** | ·   VAS | NR | NR | NR |
| **Vercruyssen et al. (2016)** | ·   McGill Pain Questionnaire | Melzack, Ronald. 1975. “The McGill Pain Questionnaire: Major Properties and Scoring Methods.” Pain 1(3):277–99. doi: 10.1016/0304-3959(75)90044-5. Melzack, Ronald. 2005. “The McGill Pain Questionnaire: From Description to Measurement.” Anesthesiology 103(1):199–202. doi: 10.1097/00000542-200507000-00028. | **No** | **No** |
|  | ·   Health-related quality of life (HRQOL) instrument | Shugars, D. A., K. Benson, R. P. White, K. N. Simpson, and J. D. Bader. 1996. “Developing a Measure of Patient Perceptions of Short-Term Outcomes of Third Molar Surgery.” Journal of Oral and Maxillofacial Surgery: Official Journal of the American Association of Oral and Maxillofacial Surgeons 54(12):1402–8. doi: 10.1016/s0278-2391(96)90253-0. | **No** | **No** |
|  | ·   VAS | Nkenke, Emeka, Stefan Eitner, Martin Radespiel-Tröger, Eleftherios Vairaktaris, Friedrich Wilhelm Neukam, and Matthias Fenner. 2007. “Patient-Centred Outcomes Comparing Transmucosal Implant Placement with an Open Approach in the Maxilla: A Prospective, Non-Randomized Pilot Study.” Clinical Oral Implants Research 18(2):197–203. doi: 10.1111/j.1600-0501.2006.01335.x. | **No** | **No** |
| **Peñarrocha-Oltra et al. (2014)** | ·   VAS | McGrath, Colman, Otto Lam, and Niklaus Lang. 2012. “An Evidence-Based Review of Patient-Reported Outcome Measures in Dental Implant Research among Dentate Subjects.” Journal of Clinical Periodontology 39 Suppl 12:193–201. doi: 10.1111/j.1600-051X.2011.01841.x. | **No** | **No** |

NR= Not Reported
