# Supplementary material for: Patient‐ and Clinician‐Reported Outcomes and Outcome Measures Evaluating Timing of Implant Loading in the Edentulous Maxilla: A Systematic Review of Prospective Studies
Source: Clin Oral Implants Res. 2026 Feb 24;37(Suppl 30):S332–45. doi: 10.1111/clr.14451 (PMC12930122; doi:10.1111/clr.14451)
Supplement: Supplementary file 5 — Table S5. CROs and CROMs references. [file CLR-37-S332-s003.docx]

Table S5 – ClinROs references

| **Study** | ClinROs | References for ClinROs cited in the study |
| --- | --- | --- |
| **Marković et al. (2022)** | -Implant stability | -Lages FS, Douglas-de Oliveira DW, Costa FO. Relationship between  implant stability measurements obtained by insertion torque and resonance  frequency analysis: a systematic review. Clin Implant Dent Relat Res. 2018;20:  26–33.  -Norton MR. Resonance frequency analysis: agreement and corre-  lation of implant stability quotients between three commercially available  instruments. Int J Oral Maxillofac Implants. 2018. doi: 10.11607/jomi.6964. |
|  | -VAS for clinician satisfaction  -Insertion Torque  - Surgical and prosthetic complications | NR |
| Montero et al. (2021) | - Occlusal force/area  - EMG activity | NR |
| **Bernard et al. (2019)** | -Bone Loss measure by ImageJ software | Abràmoff MD, Magalhães PJ, Ram SJ. Image processing with imageJ. Bio-  photonics Int 2004;11:36-41. |
|  | -Probing Depths | NR |
|  | -Bleeding on Probing | Weinberg MA, Hassan H. Bleeding on probing: what does it mean. Gen Dent  2012;60:271-8. |
|  | -Plaque index | Orban B. Gemodiﬁceerde Navy Plaque index. In: Parodontale indices II. 1972. p. 8. |
| **Vercruyssen et al. (2016)** | -Time duration of the procedure | NR |
|  | -Accuracy of implant Placement | -Maes, F., Collignon, A., Vandermeulen, D., Marchal, G. & Suetens, P. (1997) Multimodality image registration by maximization of mutual information. IEEE Transactions on Medical Imaging 16: 187–198.  - Vercruyssen M, Coucke W, Naert I, Jacobs R, Teughels W, Quirynen M. Depth and lateral deviations in guided implant surgery: an RCT comparing guided surgery with mental navigation or the use of a pilot-drill template. Clin Oral Implants Res. 2015 Nov;26(11):1315-20. doi: 10.1111/clr.12460. |
| **Peñarrocha-Oltra et al. (2014)** | NR | NR |

NR= Not Reported
